# Progress in Obstetrics and Gynæcology

**Published:** 1898-08-06

**Authors:** 


### Progress in Obstetrics and Gynæcology.

**Inversion of the Uterus.**—Three interesting cases of this complication are reported by different writers. Switalski[1] reports one in which the patient, a multipara, aged 36, aborted at the fifth month. The placenta was expelled after some hours, during which much bleeding took place. Repeated hæmorrhages, occurring later, led to further advice, when the uterus was found to be completely inverted, the fundus lying in the vagina, while about half an inch of much-thinned cervix occupied the normal position; the external os was about an inch in diameter. The diagnosis was rendered complete by the feeling of a cup at the upper end of the uterus on examination per rectum. Manual reposition under chloroform, after drawing down the cervix, was attempted, but caused a laceration of the anterior portion of the cervix, which extended as high as the internal os and caused separation of the bladder. The posterior cul de sac was then opened and median incision of the posterior wall of the uterus made, which extended from one inch above the os externum to one inch from the fundus. Reinversion of the uterus was thus rendered easy, and after its completion the incisions in the uterine wall and the posterior fornix were sutured, and the lacerated cervix repaired. The patient made a good recovery. On examination of a portion of the uterine wall, removed for the purpose, degenerative changes were found; these changes probably led to the laceration of the cervix, a careful examination of which is necessary before attempting manual reposition. In a case such as the foregoing, when the uterine wall is unhealthy, gradual reposition would probably be less dangerous. This complication is exceedingly rare after abortion. The author could only find records of two similar cases. Raparewski[2] was called to see a woman whom he found to be pulseless, lying in a pool of blood, and with a mass as big as a child's head projecting from the vulva. He found that the uterus was completely inverted, and that the cause was traction on the cord for the removal of an adherent placenta, which still was partially adherent to the uterine wall, to which also adhered the membranes. On repressing the uterus into the vagina sudden reduction occurred without further manipulation. The patient, however, died in a few hours, apparently from the combined effects of shock and hæmorrhage.

Schacht[3] was called to see a primipara in whom strong after-pains with retention of urine had occurred after a forceps delivery. The after-pains were attributed to the retention of urine, which persisted for eight days, during which time catheterisation was necessary. There was free, but not excessive loss during the first ten days; the colour then became dirty brown and odour offensive; the temperature had varied between 99 deg. and 100 4 deg. during the whole of the fortnight. An examination showed that the fundus in a very œdematous condition occupied the vagina. There was no local tenderness or pain on examination. Reduction was effected under anæsthesia after some little difficulty, and the patient made a good recovery, the discharge becoming non-offensive, and the temperature sinking to normal. The occurrence of the severe after-pain and the retention of urine, together with the œdema of the fundus, point to the inversion having taken place within a short time after delivery, but whether it followed the expulsion of the placenta, or even on the following day, when the after-pain commenced, seems uncertain. The absence of the fundus from the hypogastrium after delivery seems to have been unnoticed, although at the time reduction was effected this was obvious: the omission of this easy investigation seems to have been to a great extent the cause of the failure to diagnose the condition earlier, when reduction would probably have been considerably easier. After reduction the uterus was douched and packed with iodoform gauze.

**Rupture of the Uterus.**—An instance of this accident occurring during pregnancy is reported by Jellinghaus;[4] the patient was in her ninth pregnancy, and had suffered from hæmorrhage due to adherent placenta at each of her previous confinements; in each case manual removal of the placenta proved necessary. In the sixth month of her ninth pregnancy she had some bleeding and pain following on a fall from a window. It was found that she was miscarrying, the progress of the miscarriage being very slow. A laxative was administered, and it was noticed after a few watery evacuations that the uterus was tense and the abdomen distended, with dulness in its lower portion. The dulness and distension of the abdomen gradually increased with increasing pain. On account of these symptoms the abdomen was opened, when it was found that rupture of the uterus had occurred. The child was removed, and Porro's amputation of the uterus performed. The patient recovered after some time. The uterine walls were found to be excessively thin, having, no doubt, been weakened by the repeated pregnancies and possibly by the manual removal of the placenta. The latter fact would appear to indicate the presence of some disease of the endometrium. The thinning of the uterine wall might be due to this instead of to the removal of the placenta by the hand, which latter could not be expected to have any permanent effect.

Healey[5] reports two cases of spontaneous rupture of the uterus during labour. In the first case he was called to a multipara, whose previous labours had been tedious. On his arrival he found the patient in strong labour, the head high and not engaged. The liquor amnii had escaped several hours before. The head making no advance in spite of strong pains, he advised instrumental delivery, but did not succeed in obtaining the consent of the friends until after the lapse of three hours. At this point sudden collapse of the patient occurred, and the pains ceased. Extraction was performed as rapidly as possible. The child was dead. The placenta was removed manually. On examination a rent was found in the anterior uterine wall extending half round the circumference of the lower segment. The child weighed 15 lbs. The mother died shortly after delivery, apparently from shock, so that no operation could be undertaken. In the second case he attended the patient was a multipara, and her previous labours had been normal. The membranes ruptured directly after his arrival. He found the vertex presenting in the right occipito posterior position, the head lying at the brim. The pains were strong, and rapid advance was made. An hour after the rupture of the membranes the head was distending the perineum and the occiput under the pubic arch, when sudden collapse occurred and cessation of the pains, while the head receded to the brim of the pelvis, and the body could be distinctly felt through the abdominal wall. Assistance was sent for, and the forceps applied to the head, and after considerable manœuvring the child was delivered. Great difficulty was caused by the whole of the body having escaped from the uterus into the abdominal cavity, and the consequent impaction of the shoulders on the abdominal side of the rent in the uterus. A transverse rent was found extending through two-thirds of the circumference of the lower segment, which latter formed one-fourth of the body of the uterus. The placenta was removed without difficulty, but the small intestine prolapsed through the rent during its removal. The patient died almost immediately after delivery. No ergot or other oxytocic had been administered. In neither of these cases was any operation possible, and in a large number of cases of rupture this will often be the case, since profound shock is a usual accompaniment to this accident, and is generally superadded to exhaustion due to prolonged labour. Considerable hæmorrhage, also, is likely to occur, and must be looked for carefully, as it may be very largely intraperitoneal, and so not obvious externally. The combating of the shock by the injection of ether and strychnia hypodermically and hot saline and alcohol by the rectum will in many cases tide the patient over the period of collapse until it is possible to operate without the additional shock determining the fatal event. The chief indication for operation is the evidence of free hæmorrhage or a laceration of such extent that it cannot be safely dealt with by less drastic measures.

[1] Epit. Brit. Med. Jour., April 16, 1898. [2] Indian Med. Rec., Mar. 16, 1898. [3] Amer. Med. Surg. Bulletin, Jan. 25, 1898. [4] Amer. Jour. Med. Sci., Feb., 1898. [5] Boston Med. Surg. Jour., April 21, 1898.

